# Intelligent modeling and optimization of titanium surface etching for dental implant application

**DOI:** 10.1038/s41598-022-11254-0

**Published:** 2022-05-03

**Authors:** Seyyed Mohamad Sadati Tilebon, Seyed Amirhossein Emamian, Hosseinali Ramezanpour, Hashem Yousefi, Mutlu Özcan, Seyed Morteza Naghib, Yasser Zare, Kyong Yop Rhee

**Affiliations:** 1 Surface Engineering Unit, AVITA Dental System, KFP-Dental Company, Tehran, Iran; 2 Research and Development Unit, AVITA Dental System, KFP-Dental Company, Tehran, Iran; 3 Division of Dental Biomaterials, Center for Dental and Oral Medicine, Clinic for Reconstructive Dentistry, University of Zürich, Zurich, Switzerland; 4 Nanotechnology Department, School of Advanced Technologies, Iran University of Science and Technology, P.O. Box 16846-13114, Tehran, Iran; 5 Department of Mechanical Engineering, College of Engineering, Kyung Hee University, Yongin, 446-701 Republic of Korea

**Keywords:** Biomaterials, Biomedical engineering

## Abstract

Acid-etching is one of the most popular processes for the surface treatment of dental implants. In this paper, acid-etching of commercially pure titanium (cpTi) in a 48% H_2_SO_4_ solution is investigated. The etching process time (0–8 h) and solution temperature (25–90 °C) are assumed to be the most effective operational conditions to affect the surface roughness parameters such as arithmetical mean deviation of the assessed profile on the surface (R_a_) and average of maximum peak to valley height of the surface over considered length profile (R_z_), as well as weight loss (WL) of the dental implants in etching process. For the first time, three multilayer perceptron artificial neural network (MLP-ANN) with two hidden layers was optimized to predict R_a_, R_z_, and WL. MLP is a feedforward class of ANN and ANN model that involves computations and mathematics which simulate the human–brain processes. The ANN models can properly predict R_a_, R_z_, and WL variations during etching as a function of process temperature and time. Moreover, WL can be increased to achieve a high Ra. At WL = 0, R_a_ of 0.5 μm is obtained, whereas R_a_ increases to 2 μm at WL = 0.78 μg/cm^2^. Also, ANN model was fed into a nonlinear sorting genetic algorithm (NSGA-II) to establish the optimization process and the ability of this method has been proven to predict the optimized etching conditions.

## Introduction

Creating rough surfaces on medical implants has been shown to improve their bio-integration resulting from increased bone tissue production at the modified surfaces^1–4^. Many studies have shown that undesirable fibrous tissues tend to form on smooth surfaces, while rougher surfaces promote the formation of solid bone tissue^5,6^ conducted a long-term study of bone integration with implants^7,8^. Bone is generated in a multistep process wherein osteoclast cells selectively remove tissue to form pits (lacunae), and the osteoblast cells, in turn, collect and form the bone matrix tissues^9^. As osteoclasts cannot interact with implant materials, such as titanium (Ti), osteoblast bonding can be inhibited if an implant’s surface does not possess micro-, meso-, ornano-scale structures similar to those created by osteoclasts^10^.

Usually, surface roughness is checked with some parameters like R_a_, R_z_, and S_a_. R_a_ is a 1-D parameter and is defined as arithmetical mean deviation of the assessed profile^11^. For measurement of R_a_, a very thin detector tip moves in a horizontal linear direction over the surface of the sample (in contact or noncontact mode), that vertical moves will be reported^12,13^. R_z_ is maximum peak to valley height of the same profile, that is studied for R_a_ measurement^12^. S_a_ is an areal roughness parameter that is detected from a 2-D surface (unlike Ra that is detected from a linear path)^11^. Based on definitions, R_a_ and S_a_ are very close to each other and choosing one of them is based on measurement equipment (some of them report R_a_ and some other report S_a_)^11,12^.

Etching is one of the most popular processes used to enhance surface roughness and improve other properties of the surface. It is responsible for enhancing a dental implant’s contact with bone and can specify the strength of the implant’s contact with the surface^11–15^ and bone response^16–20^. Currently, a wide range of commercial dental implants is available in the market. These implants have differing properties, such as the implant’s core, geometric specifications, and surface characteristics. Some commercial brands, such as Ospol (Hollviken, Sweden), with S_a_ = 0.26 μm, have a smooth surface (S_a_ < 1 μm is considered smooth)^21^. On the other hand, other commercial implants, such as SLA (Standard Plus; ITI Straumann, Basel, Switzerland; S_a_ = 1.7 μm), Ankylos (DentsplyFriadent, Menheim, Germany; S_a_ = 1.55 μm), Frialit (DentsplyFriadent, Menheim, Germany; S_a_ = 1.79 μm), and Promote (Screwline, calmog, Basel, Switzerland; S_a_ = 1.30 μm), are moderately rough (with S_a_ in the range of 1–2 μm)^22,23^. Additionally, some other surfaces, such as the Kohno HRPS and Kohno DES HRPS (Sweden & Martina, Due Carrare, Italy; S_a_ = 3.11 and 3.16 respectively), have the highest degree of roughness (surfaces with S_a_ > 2 are considered maximally rough)^22,23^.

In 2010, Elias^24^ reported that acid-etched implants have a more homogeneous surface compared to machined surfaces. In addition, the acid etching process, when applied as a pretreatment for anodizing the dental implants (or other processes), provides homogeneous roughness, a large active surface area, and improves bio-adhesion. Grizon and coworkers^25^ conducted a long-term study to investigate the enhancement of bone-implant integration with increased surface roughness. No significant differences were observed for the two types of implants between 3–6 months. At 12 and 18 months, the bone volume and contact interface were still increasing, and the implants with R_a_ = 0.206 µm were associated with higher values than the smoother implants (R_a_ = 0.160). Many similar studies, such as one by Fouziya et al.^26^ reported that, smoother surfaces require a longer time for osseointegration and prosthetic loading.

**Figure 1:**
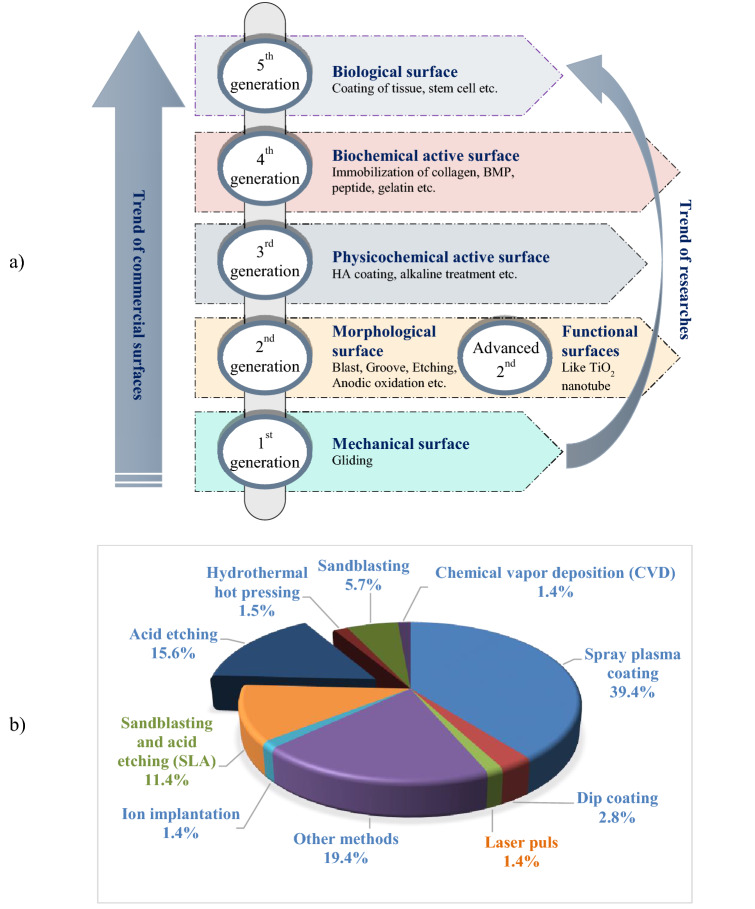
(**a**) Different generations of dental implant surface modifications, and (**b**) commonly used surface modification methods for titanium-based dental implants.

The trends in dental implant surface modifications can be classified into the five generations shown in Fig. 1a. Etching was part of the first generation of methods for improving machined surfaces with mechanical treatment. Despite all the improvements to surface modification methods, etching is still widely used in commercial systems, either directly as a final surface modification or in combination with other methods. Based on Fig. 1b, after the plasma spray coating process, etching, with a more than 15% usage rate, is the second most used method based on published reports. In addition, several of the other methods, like sandblasting and sandblasting plus acid etching (SLA), use etching as a treatment method^27^.

Acid etched has a lower risk of implant surface contamination than blasting since there are no particle remnants on the surface^28^. This surface enhances the migration and retention of osteogenic cells. There are variations in acid etching methods between the different manufacturers based on acid concentration, process time, and temperatures. Acid etching forms micropits on the implant surface^29^ and titanium hydrides that are replaced by oxygen, slowing the transformation of the implant surface. In addition, nanosized titanium particles are formed on the surface that favor the adhesion of proteins through surface nano-roughness features^30^. Acid-etched surfaces show more bone apposition, increasing the interfacial strength as calculated by removal torque^31–33^ or push-out tests^14,34^. Moreover, etching the dental implant surfaces leads to a reduction in the healing time in the mandible and the maxilla at 6–8 weeks (from 3–6 months)^35–38^.

Mathematical models for etching process can lead to choosing suitable operation condition and having desirable surface for dental implants. Between different methods of modelling, artificial neural networks (ANN) have many advantages over others and intelligent modelling using experimental datasets for model configuration have more reliability. ANNs are a technology based on the studies of the nervous system and brain. These networks emulate a biological neural network but they use a reduced set of concepts from biological neural systems. Specifically, ANN models simulate the electrical activity of the brain and nervous system. Processing elements (also known as either a neuron or perceptron) are connected to other processing elements. Typically, the neuron is arranged in a layer or vector with the output of one layer, serving as the input to the next layer and possibly other layers. A neuron may be connected to all or a subset of the neuron in the subsequent layer. Weighted data signals entering a neuron simulate the electrical excitation of a nerve cell and consequently, the transference of information within the network or brain. Output of this data transfer between neurons, is processing on the data and prediction of output. ANN model should be optimized by training, validation and test^39–42^. Despite all advantages of ANN, a reliable model is needed to correct experimental datasets and collection of suitable data in appropriate number, that is costly and time consuming.

Numerical study of surface characteristics for dental implant application, is relatively new field of study. In 2020, Kohler et al.^43^ reported a numerical model for titanium acid etching. Their model assumed R_a_ to be the only determining parameter. Weight loss as a parameter of geometrical limitations should be fixed in an acceptable range (based on quality control protocols). Intelligent modeling with artificial neural networks (ANN) can address the drawbacks of the other methods of modelling such as the inability to fit large amounts of data. Therefore, multilayer perceptron (MLP) ANN is used to model the etching process in 48% H_2_SO_4_ solution. In the following, nonlinear sorting genetic algorithm (NSGA-II) optimization method is utilized to arrive at the optimal conditions for the process.

## Materials and methods

In this study, MLP-ANN was used to investigate the surface characteristics including the R_a_, R_z_, and WL when etching solution temperature and etching process time varies. A MLP-ANN with two hidden layers was used for achievement to the optimized topology of ANN, 1 to 5 neurons were tested in each hidden layer. Finally, ANN structure with lowest MSE was proposed as the best ANN model and fed to the NSGA-II for process optimization.

Ban et al.^44^ investigated etching time and etching solution media temperature effects on surface characteristics. Data were feeding into ANN models. The following equation [Eq. (1)] was used to normalize input variables in rang of [–1, + 1]:

1 $${\text{X}}_{{\text{i}}} = 2 \times \frac{{{\text{x}}_{{\text{i}}} - {\text{x}}_{\min } }}{{{\text{x}}_{\max } - {\text{x}}_{\min } }} - 1,$$

where Xi is a normalized value of input variable xi, x_min_ is minimum value of target functions and x_max_ is the maximum one^39,45^.

An in-house computational code was developed in this study that can conceptually search for best ANN configurations with dividing data into ‘training’, ‘validation’, and 'test’ sets. Accordingly, 70% of data was randomly chosen to train the model. On the other hand, 15% of data was fed to ANN model for validation. Finally, the rest of the data was used for testing the configured ANN structure. Although there is no rigid rule to find the appropriate number of neurons in the hidden layers, the complexity of the relationship between inputs and outputs plays a key role^46,47^. Different combinations of neurons (one to five neurons in each hidden layer) in two hidden layers were tested for choosing the best configuration with a minimized error. The function defined through Eq. (2) was applied in output, and hidden layers were used as activation transfer function^40,48^:

2 $${\text{f (x)}} = \frac{{{\text{e}}^{{\text{x}}} - {\text{e}}^{{ - {\text{x}}}} }}{{{\text{e}}^{{\text{x}}} + {\text{e}}^{{ - {\text{x}}}} }}.$$

**Figure 2:**
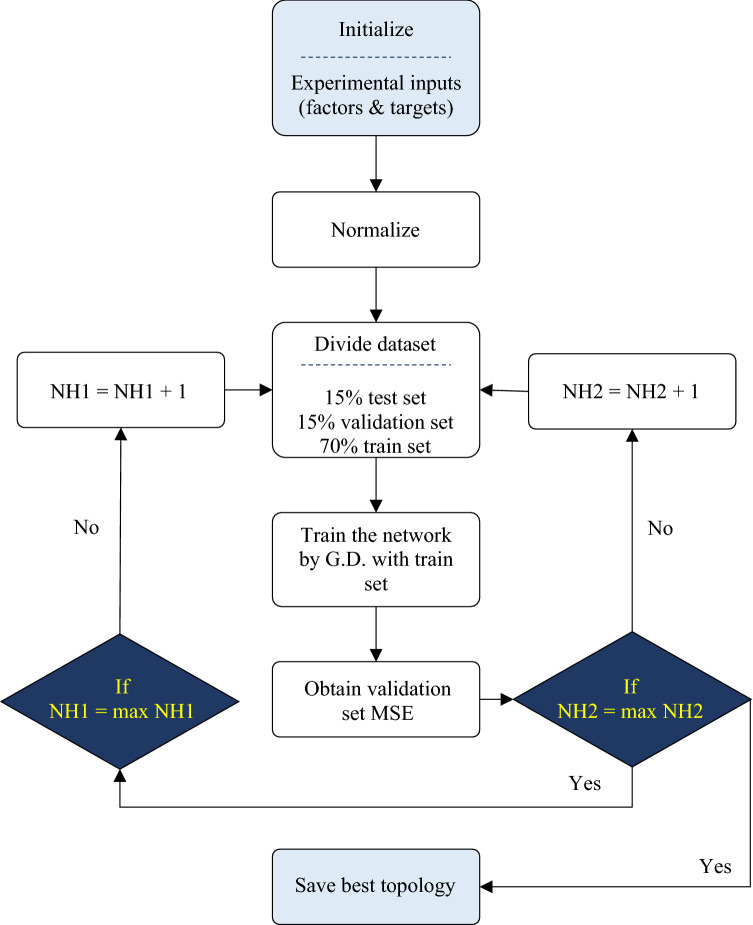
Modeling flowchart based on ANN method.

A well-organized in-house code based on the flowchart illustrated in Fig. 2 was used to model R_a_, R_z_, and WL (three different models). A gradient descent (G.D.) method was used to model optimization parameters and find the best biases and weights to match the input and output variables.

Number of neurons in hidden layer 1 and hidden layer 2 varied in range of 1 to 5 (max NH1 and max NH2 is equal to 5). Model accuracy for response prediction was measured by mean of squared error (MSE) criterion^49,50^ as noted in Eq. (3)^39,46,47,51^.

3 $${\text{MSE}} = \frac{{1}}{{\text{n}}}\sum\limits_{{{\text{i}} = {1}}}^{{\text{n}}} {{\text{ (Y}}_{{\text{i}}} - {\overline{\text{Y}}}_{{\text{i}}} )^{2} } ,$$

where n is the number of samples, Y_i_ and $\overline{{\mathrm{Y} }_{\mathrm{i}}}$ are experimental and predicted value of response for sample i.

**Figure 3:**
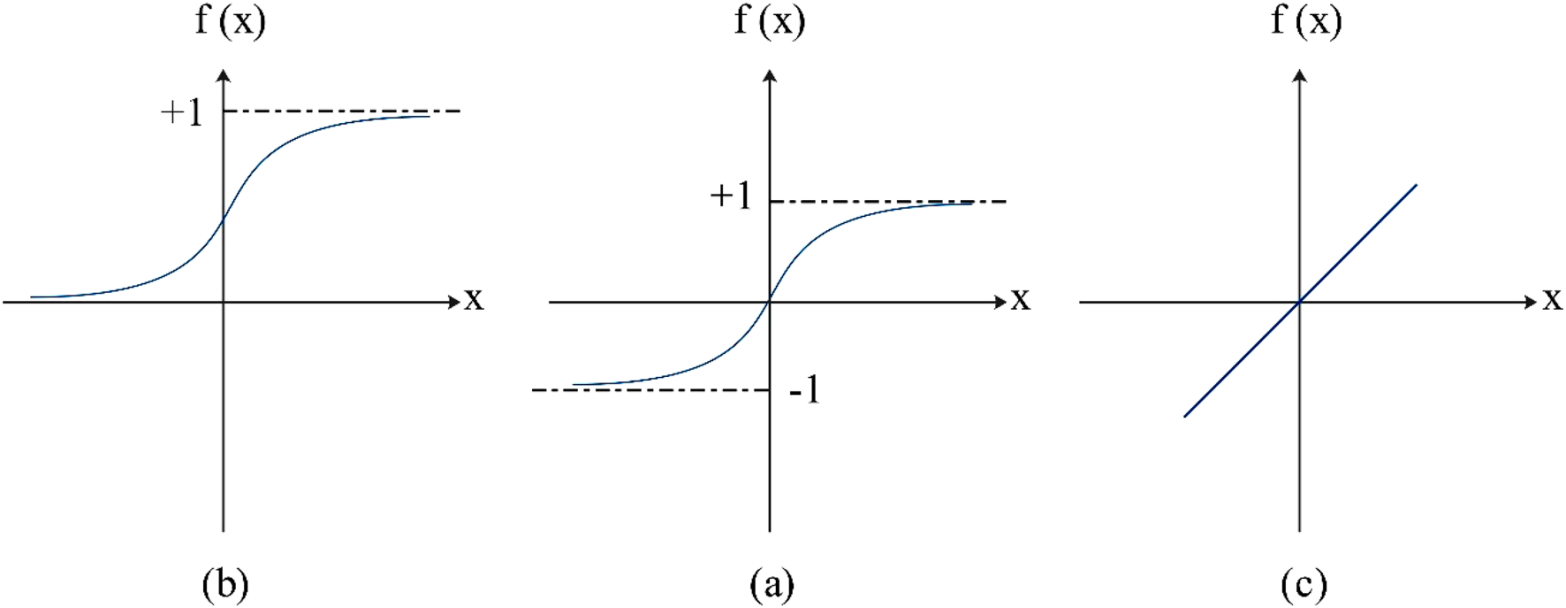
Typical transfer functions (**a**) Logsig, (**b**) Tansig, and (**c**) Pureline.

To reach the desirable model the inputs must be normalized and also activation transfer function should be used for output and hidden layers. Several types of activation transfer function are utilized. Three of these functionsaremore important and applicable that shown in Fig. 3; Pureline [Eq. (4)], Logsig [Eq. (5)], and Tansig [Eq. (7)].

- Linear transfer function (Purelin)
  4 $${\text{f (x)}} = {\text{s}}$$
  This transfer function is regularly employed in the output layer. The primary interest of MLPs resides in their nonlinear sigmoid function (such as logsig and tansig) principally used in their hidden layers.
- Log-Sigmoid transfer function (Logsig)
  5 $${\text{f (x)}} = \frac{{1}}{{{1} + {\text{e}}^{{ - {\text{x}}}} }},$$
  which is easily differentiable, and frequently used as a nonlinear transfer function for engineering applications. However, because it is limited between 0 and 1, its linearly transformed type is used instead. It is recognized as the bipolar transfer function [Eq. (6)]:
  6 $$f(x) = \frac{2}{{1 + e^{ - x} }} - 1$$
- Hyperbolic Tangent Sigmoid (Tansig)
  7 $$f(x) = \frac{{e^{x} - e^{ - x} }}{{e^{x} + e^{ - x} }},$$
  which is very alike in kind and shares many mathematical properties with the bipolar transfer function and it is bounded between − 1 and + 1. It is too often employed in engineering application.

In the light of the above procedure, three ANN modelswere optimized to investigate the effective parameters on surface properties (R_a_, R_z_, and WL). In the next step, for the multi-objective optimization of the etching process in 48% H_2_SO_4_ solution, a Non-Dominated Sorting Genetic Algorithm-II (NSGA-II) was utilized. For the production of chromosomes, the value of each gene was randomly chosen in view of the values of variables given in Table 1. The code was then implemented to find the fitness of each chromosome to optimize surface properties simultaneously. After giving pairs of chromosomes were compared together, they were isolated from the remainder, as the first Pareto front. This operation was surveyed to obtain Pareto fronts 2, 3, etc. Pareto front n was assigned to the chromosome that has been dominated (n − 1) times. We also used crowding distance (C.D.) values, as the second criterion for optimization, was to sort pareto fronts [Eq. (8)]:

8 $${\text{C.D.}}\,{\text{(i)}} = \sum\limits_{{{\text{x}} = {1}}}^{{\text{N}}} {\frac{{{\text{d}}_{{\text{e}}} {\text{(i)}}}}{{{\Delta }_{{\text{x}}} {\text{(i)}}}}}$$

where $${\text{d}}_{{\text{x}}} {\text{(i)}} = {\text{| F}}_{{\text{x}}} {\text{ (i}} + {1)} - {\text{F}}_{{\text{x}}} {\text{(i}} - {1) |}$$ and $${\Delta }_{{\text{x}}} {\text{ (i)}} = {\text{| max F}}_{{\text{x}}} - {\text{min F}}_{{\text{x}}} { |}$$.

**Table 1:** The values of parameters used for multi-objective optimization by G.A. approaches.

Optimization parameter	Value
Initial population size	25
Crossover mechanism	Arithmetic
Crossover rate	70%
Mutation mechanism	Polynomial
Mutation rate	40%
Selection mechanism	Ternary tournament selection
Maximum iteration number	50

**Figure 4:**
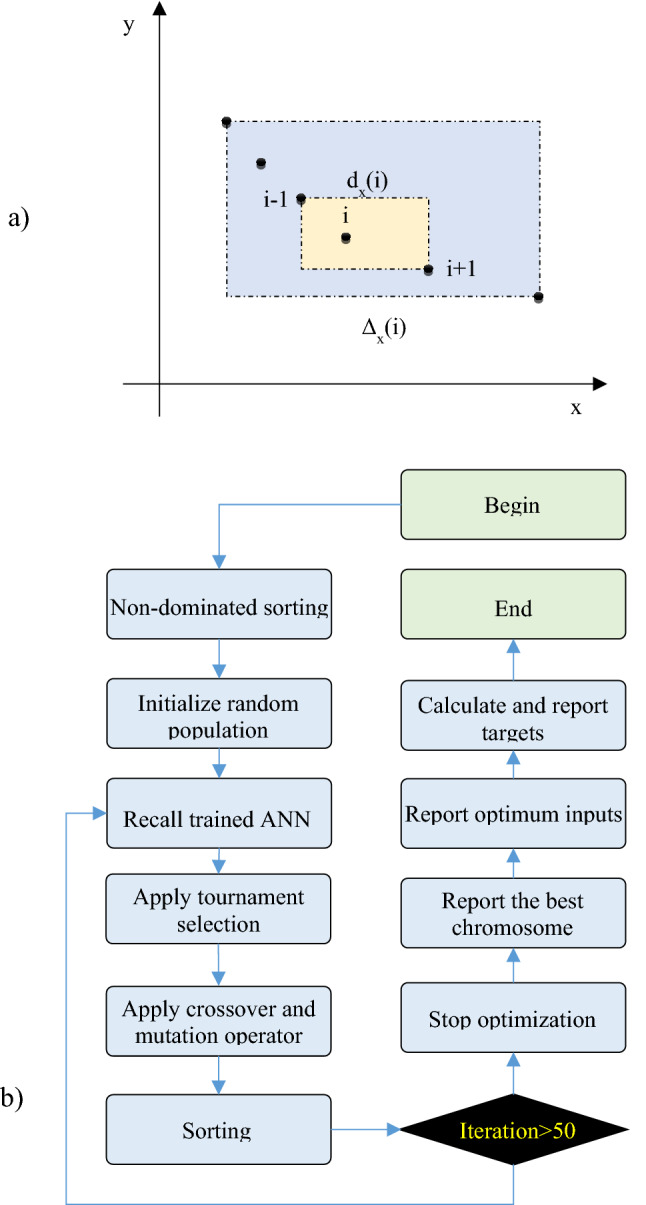
(**a**) Crowding distance calculation parameters for sample i, and (**b**) flowchart of NSGA-II based optimization.

In Eq. (8), N is the number of objective functions, C.D. (i) is the crowding distance of the chromosome i, and d_x_ (i) and Δ_x_ (i) are based on the objective function x, as shown in Fig. 4a. After estimating the degree of fitness based on primary and secondary constraints, chromosomes were sorted according to their favorability. The best chromosomes were chosen as the first Pareto front, followed by selecting, pairing, reproducing, and muting. The fitness of the parents and of the children of the new generation was revisited by utilizing a non-dominated sorting algorithm. Table 1 gives the values of parameters employed to optimizethesurface properties based on the NSGA-II evolutionary algorithm (Fig. 4b).

## Results

### ANN-based model evaluation

#### R_a_ ANN structure configuration and model validation

**Figure 5:**
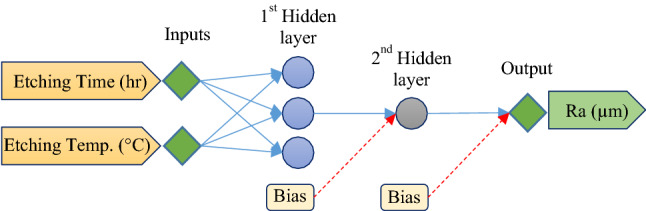
A schematic of the ANN model structure for R_a_ prediction based on etching time and etching ambient temperature.

The ANN-based model evaluation for the R_a_ was completed in the first step. As shown in Fig. 5, an ANN model with two hidden layers (three neurons in the first hidden layer and one neuron in the second hidden layer) was the best structure for R_a_ prediction based on temperature (°C) and time (h) variation. Different combinations of Tansig and Logsig transfer functions were approved, and the results showed that the Tansig transfer function for both hidden layers performed best. The correlation coefficient (R) for the fixed ANN model, was 0.9892 that is very close to 1; this indicated that the model is reliable.

**Figure 6:**
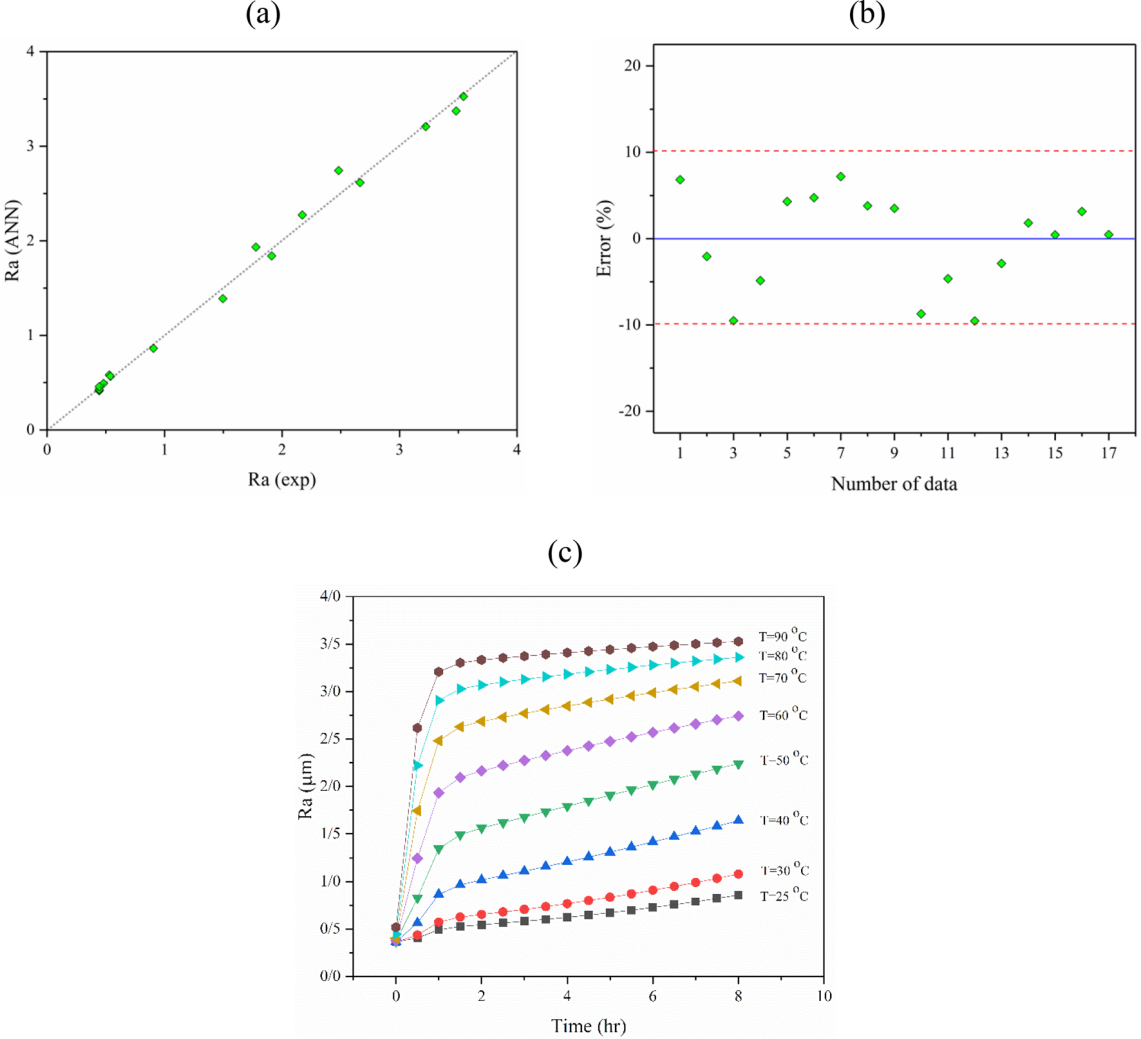
(**a**) Quality line, (**b**) the error values for experimental data and ANN model (R = 0.9892) outputs of R_a_, and (**c**) the effect of time and temperature on the R_a_ of the titanium surface after etching with an H_2_SO_4_ etchant solution.

In the following, the accuracy of the model was studied by the quality line. As illustrated in section (a) of Fig. 6, the model fit is perfect if all the predicted data equals the experimental data. Therefore, the model has high accuracy if all data fall close to the y = x line. The ANN model predicted the experimental data with less than 10% error (see Fig. 6). As a result, the configured model was used to predict R_a_ under different operational conditions in the time range of 0 to 8 h and different temperatures (25, 30, 40, 50, 60, 70, 80, and 90 °C), as presented in Fig. 6c.

#### R_z_ ANN structure configuration and model validation

**Figure 7:**
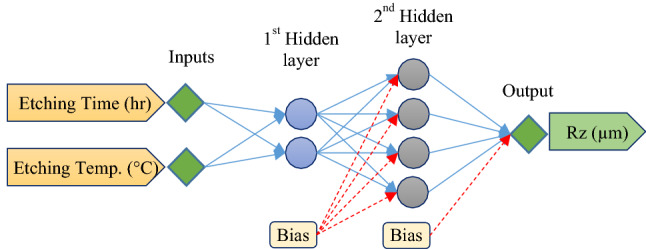
A schematic ANN model structure for R_z_ prediction based on etching time and solution temperature.

The second ANN model was configured to predict R_z_ on the titanium surface during acid etching with 48% H_2_SO_4_ with etching time and solution temperature variation. The best ANN structure was obtained by an ANN model with two neurons and four neurons in the first and second hidden layers, respectively. The best transfer function for the first hidden layer was Logsig, while Tansig emerged as the best transfer function for the second hidden layer. A schematic structure of the optimized ANN model is presented in Fig. 7.

**Figure 8:**
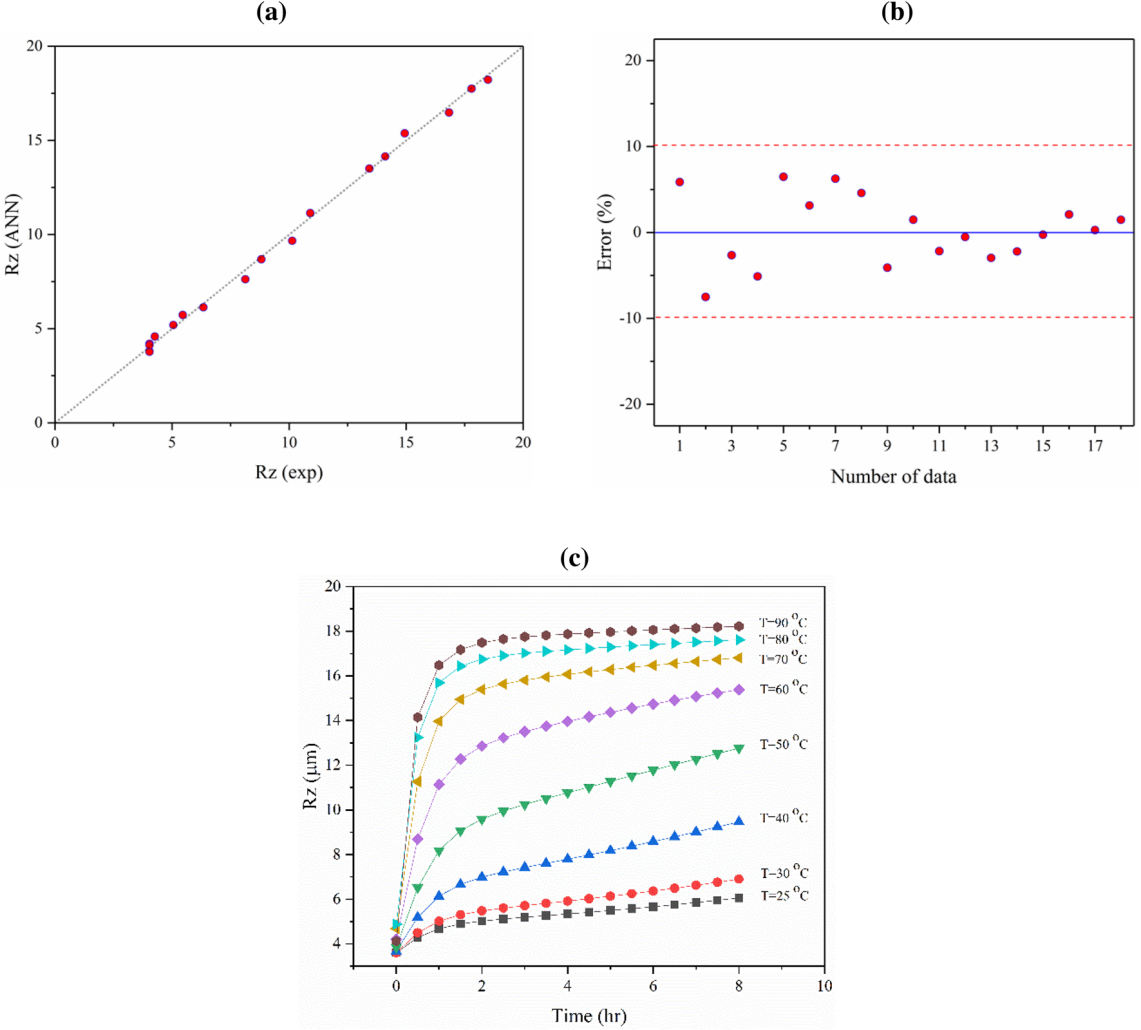
(**a**) Quality line, (**b**) the error values for experimental data and ANN model (R = 9970) outputs of R_z_, and (**c**) the effect of time and temperature variation on the R_z_ of the titanium surface after etching with an H_2_SO_4_ etchant solution.

The ANN model accuracy for R_z_ prediction was further evaluated by calculating R, error, and the quality line. The optimized ANN model for R_z_ prediction had a correlation coefficient of about 0.9970. The quality line and error diagram showed that the chosen ANN model predicted the experimental data with suitable accuracy (see Fig. 8a,b). As shown in the error diagram, all data was predicted with minimal error (less than 10%). The validated model was then used to calculate R_z_ under different conditions. The pattern of R_z_ variation based on etching time in different etching media temperatures (25, 30, 40, 50, 60, 70, 80, and 90 °C) is shown in Fig. 8c.

#### WL ANN structure configuration and model validation

**Figure 9:**
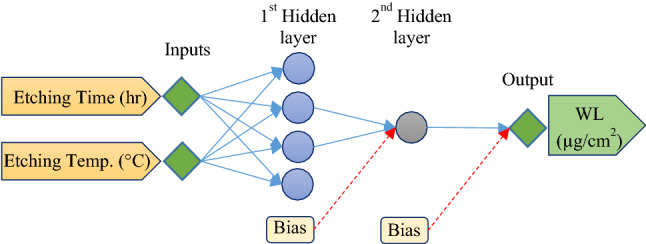
A schematic ANN model structure for WL prediction based on etching time and solution temperature.

**Figure 10:**
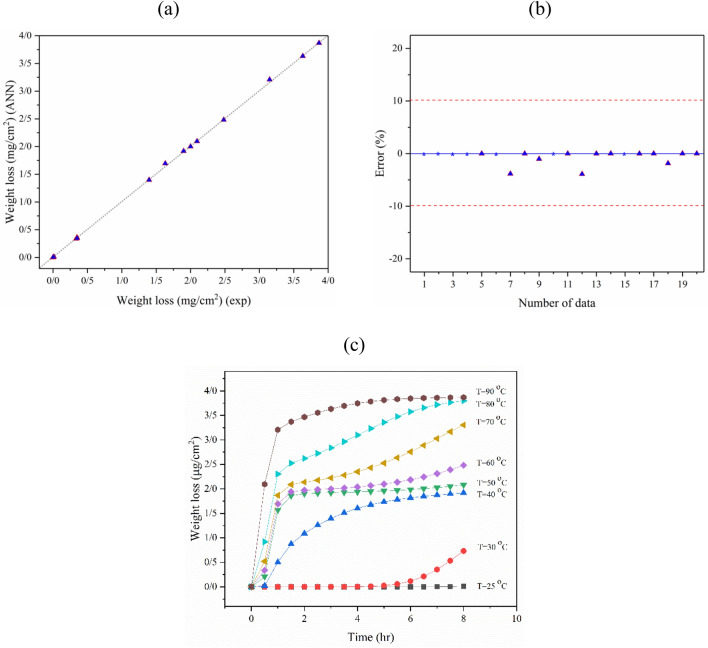
(**a**) Quality line, (**b**) the error values for experimental data and ANN model (R = 0.9991) outputs of WL (error for some points was unaccountable because of zero experimental values), and (**c**) the effect of time and temperature on the WL of the titanium surface after etching with an H_2_SO_4_ etchant solution.

The ANN model was configured for experimental WL data during the etching of titanium in 48% H_2_SO_4_. In this step, an ANN model with four neurons in hidden layer 1 and one neuron in hidden layer 2 achieved the best topology, where Tansig-Tansig was the combination of transfer functions for the first and second hidden layers. A schematic of the optimized ANN topology is illustrated in Fig. 9. Comparison of experimental data with ANN-based WL predictions showed good accuracy with a correlation coefficient of 0.9991. The quality line and prediction error are presented in Fig. 10a,b. Finally, the variation of titanium WL in the H_2_SO_4_-based etching process under different etching times and solution temperatures is shown in Fig. 10c.

### NSGA-II based optimization

#### Optimization of R_a_ and WL

As noted previously, WL and R_a_ are the two main responses to the etching process. WL should be minimized because of geometric limitations. On the other hand, studies showed that a higher R_a_ can improve an implant’s survival rate. Higher removal torque^52^ is accessible through higher R_a_, while higher surface roughness is the main factor responsible for enhancing surface area. Higher surface areas can improve the chances of bone cell growth on the titanium implant surfaces^27,53^. Therefore, the main goal of the etching process is to increase the surface roughness and decrease WL simultaneously. Unfortunately, there is a tradeoff between WL and surface roughness. Higher surface roughness is achieved at longer etching times and higher etchant temperatures, but these same conditions can increase WL. High temperatures and extended etching times can damage the substrate and change its geometric parameters.

**Figure 11:**
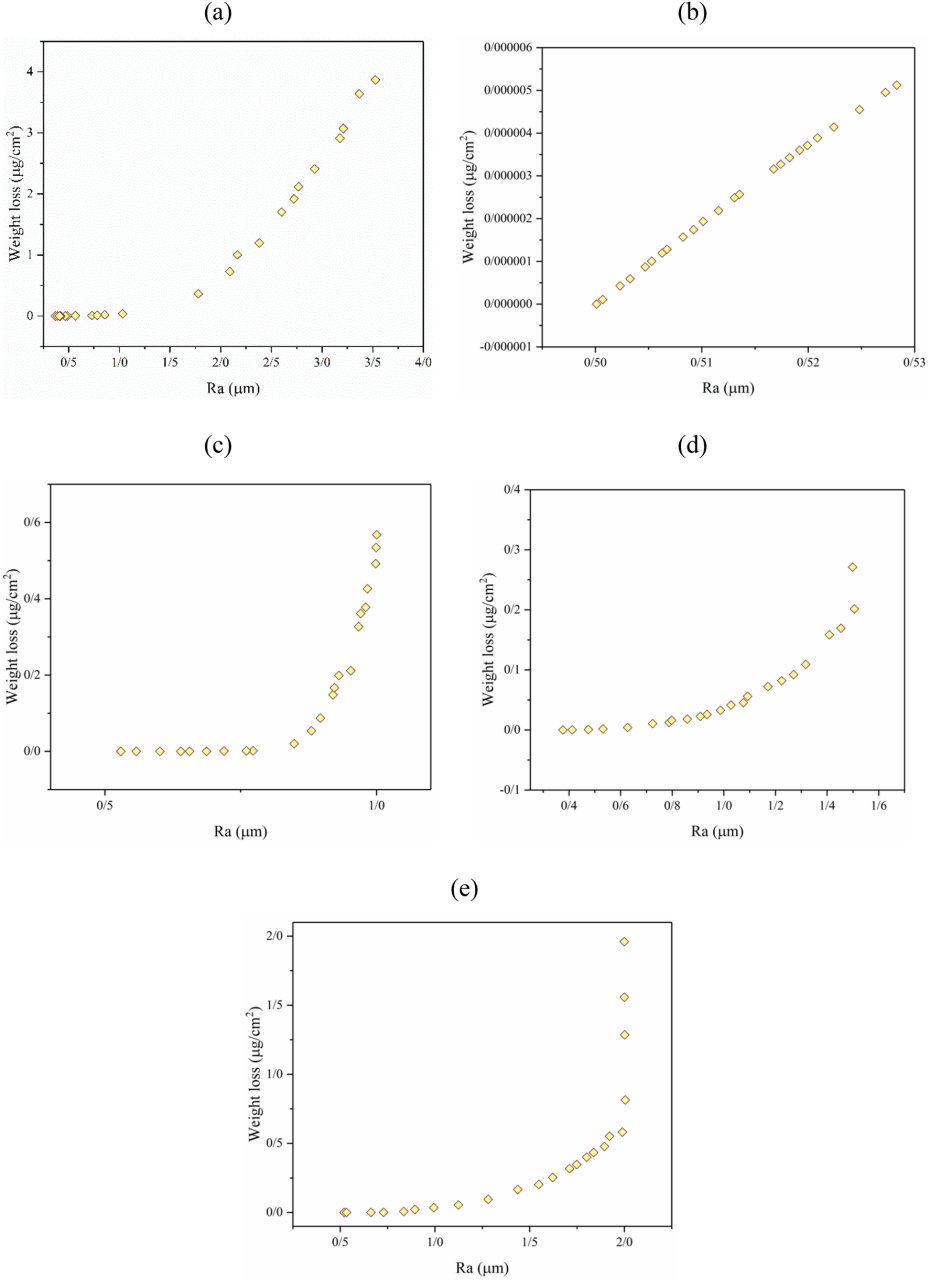
Multi-objective optimization of R_a_ and WL in the etching of titanium to (**a**) maximized R_a_ and minimized WL, (**b**) fixed R_a_ = 0.5 and WL minimized, (**c**) fixed R_a_ = 1 and WL minimized, (**d**) fixed R_a_ = 1.5 and WL minimized, and (**e**) fixed R_a_ = 2 µm and WL minimized.

The surfaces of commercial dental implants have different surface roughnesses in the range of 0.5 to less than 4 µm^21,54,55^. Surfaces with a roughness level higher than 2 µm are very limited and commonly produced by special processes, such as laser-based surface treatment methods^55,56^. On the other hand, machined surfaces of titanium-based implants are not perfectly smooth. Machined surfaces have a roughness of about 0.5 µm. On the commercial scale, surface roughnesses in the range of 0.5–2 µm are common. Therefore, multi-objective optimization was performed to minimize WL and maximize R_a_ (Fig. 11a), R_a_ = 0.5 (Fig. 11b), R_a_ = 1 (Fig. 11c), R_a_ = 1.5 (Fig. 11d), and R_a_ = 2 µm (Fig. 11e). In all cases, the tradeoff between R_a_ and WL is evident: higher R_a_ values lead to higher WL, and lower WL is achieved at lower R_a_.

Acid etching is a common base method for subsequent surface treatment processes, such as anodizing. Previous studies have shown that the roughness of etched surfaces is effective on the final treated surface of titanium^53,57^. Therefore, the best condition for the etching process is dependent on the treatment processes that follow it. However, some points with infinite C.D. are presented in Table 2.

**Table 2:** Some optimized points with infinite C.D. for R_a_–WL optimization.

Goal	Temperature (°C)	Time (h)	R_a_ (µm)	WL (µg/cm^2^)
R_a_ maximize	90.00	8.00	3.53	3.87
WL minimize	50.86	0.00	0.37	0.00
R_a_ = 0.5 µm	25.08	1.54	0.53	0.00
WL minimize	25.00	1.09	0.50	0.00
R_a_ = 1 µm	25.14	1.53	0.53	0.00
WL minimize	32.87	5.60	1.00	0.57
R_a_ = 1.5 µm	48.29	2.36	1.50	1.89
WL minimize	58.05	0.00	0.38	0.00
R_a_ = 2 µm	54.12	3.62	2.00	1.96
WL minimize	29.87	0.77	0.52	0.00

As noted in Table 2, higher temperatures and longer etching times are required to achieve higher R_a_ values. On the other hand, the lowest WL is achieved at the lowest process temperatures and shortest etching times. Therefore, objects with infinite C.D. can lead to a point with high R_a_ and high WL and another point with minimized WL and very low roughness (see Table 2). As noted in the previous sections, machined titanium substrates have an R_a_ of about 0.5 µm. Optimizing the ANN model at R_a_ = 0.5 µm for the lowest WL value leads to operating conditions with the lowest temperature and a process time close to 0 h. Moreover, the best route to achieving a R_a_ value of 2 µm was an operating temperature of 54.12 °C and an etching time of 3.62 h. Under these conditions, the lowest WL achieved is about 1.96 µg/cm^2^.

#### Optimization of R_a_ and R_z_

**Figure 12:**
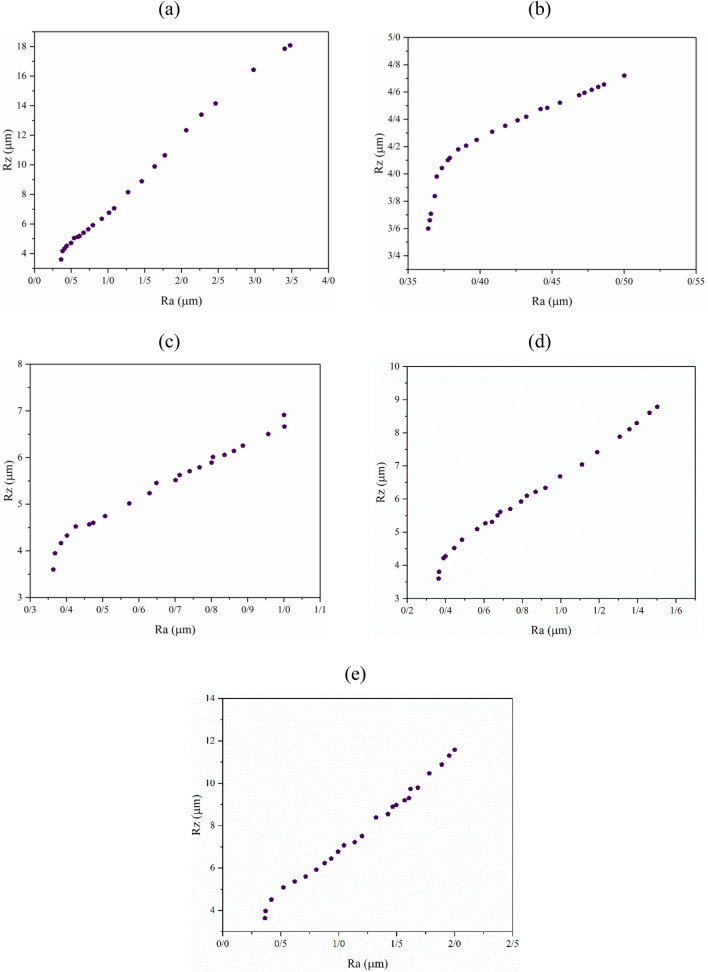
Multi-objective optimization of R_a_ and R_z_ in the etching of titanium to achieve (**a**) maximized R_a_ and minimized R_z_, (**b**) fixed R_a_ = 0.5 and minimized R_z_, (**c**) fixed R_a_ = 1 and minimized R_z_, (**d**) fixed R_a_ = 1.5 and minimized R_z_, and (**e**) fixed R_a_ = 2 µm and minimized R_z_.

Homogeneity is a target parameter for surfaces in dental implant production. Homogeneity has several effects on the characterizations of dental implant surfaces and the production process^58,59^. Achieving a perfectly reproducible product is dependent on the production of homogeneous surfaces. Variation of characteristics from point to point or product to product is very high in heterogeneous surfaces. One element of surface homogeneity is approaching the R_z_ to R_a_. It is clear that R_z_ is usually much more than R_a_. On the other hand, the difference between R_a_ and R_z_ is not a surface recognition factor as being heterogeneous. However, a surface with R_z_ close to the R_a_ value is considered more homogeneous than a surface with R_z_ far removed from the R_a_ value. All surfaces in this study have an R_z_ higher than 3 µm, which is higher than the R_a_ values. Figure 12a shows that the R_z_ of the H_2_SO_4_-etched titanium surface was altered from lower than 4 to about 18. The first objective was to minimize the R_z_ and maximize the R_a_ (Fig. 12a). The optimization to minimize R_z_ at R_a_ = 0.5, Ra = 1, R_a_ = 1.5, and R_a_ = 2 are presented in Fig. 12b–e. Some points with infinite C.D. and their operating conditions are noted in Table 3. From Table 3, we can see that the lowest achievable R_z_ is a little lower than 4 µm. At this point, a minimized R_a_ will be obtained. On the other hand, a maximized R_z_ is obtained (higher than 18 µm) under harsh process conditions that lead to the highest R_a_ (about 3.5 µm).

**Table 3:** Some optimized points with infinite C.D. for Ra–WL optimization.

Goal	Temperature (°C)	Time (h)	Ra (µm)	WL (µg/cm^2^)
R_a_ maximize	25.00	0.00	0.36	3.60
R_z_ minimize	90.00	6.26	3.48	18.08
R_a_ = 0.5 µm	25.00	0.00	0.36	3.60
R_z_ minimize	25.79	0.99	0.50	4.72
R_a_ = 1 µm	36.57	3.63	1.00	6.91
R_z_ minimize	25.00	0.00	0.36	3.60
R_a_ = 1.5 µm	25.00	0.00	0.36	3.60
R_z_ minimize	37.72	8.00	1.50	8.78
R_a_ = 2 µm	48.29	6.75	2.00	11.59
R_z_ minimize	34.60	0.00	0.36	3.64

## Discussion

The surface characteristics of dental implants affect the osseointegration process and implant survival rate. In this context, a surface treatment process that affects implant surface composition, surface roughness, and surface homogeneity is vital to developing new surfaces that facilitate osseointegration. This study provides a novel approach to using the ANN and NSGA-II to model and optimize the dental implant etching process. ANN optimizations, such as the one used in this study, have previously been used in other fields, such as surface roughness optimization of magnesium alloy machining^60^ and finish turning of AISI 4140 hardened steel^61^.

Three ANN models were developed to predict R_a_, R_z_, and WL based on H_2_SO_4_ temperature and etching duration. In agreement with previous studies, all the correlation coefficients were above 0.98. Therefore, the ANN models predicted the experimental data with a high degree of accuracy. Abbas et al.^60^ showed that when the correlation coefficient of ANN model was 0.986, high accuracy of their model in predicting surface roughness was achieved. In other study, Meddour et al.^61^ used an ANN model with correlation coefficient of 0.99 for R_a_ predicting. Therefore, the configured ANN models for R_a_ (R = 0.9892), R_z_ (R = 0.9970) and WL (R = 0.9991), are reliable. Results of this study showed that the etching process can increase the surface roughness. Lazzara et al.^62^ compared the bone response of a dual-etched surface to machined implants in the human posterior maxilla. After a healing time of six months, bone contact at the etched surface and the machined surface were 72.96% and 33.98% respectively. Additionally, a unique feature was detected at the etched surface that is bone creeping along the surface. The osteoconductive effect of the etch-textured surface over the machined surface, was particularly pronounced in the softer trabecular bone. In this type of bone, the amount of bone apposition was enhanced from 6.5 ± 10.8% for the machined surface to 59.1 ± 25.3% for the etched surface.

Low removal torque is a fundamental problem in some dental implant with relatively smooth surfaces. Low removal torque can lead to in place rotation of dental implant in prosthetic loading. Higher roughness can increase needed removal torque that can be achieved by etching process in the conditions resulted in this study. Klokkevold et al.^52^ investigated the anchoring of the etched and machined surfaces on rabbit tibia after one, two and three months. After one month, the mean removal torque of the machined surface was 6.00 ± 0.64 N cm, whereas it was 3.6 times higher for the etched surface at 21.86 ± 1.37 N cm. After two months, the difference was 3.0 times, and after three months, the etched surface required a removal torque of 27.40 ± 3.89 N cm versus 6.73 ± 0.95 N cm for the machined surface.

To achieve a homogenous surface, the optimization conditions were needed to minimize R_z_ and maximize R_a_. The conditions to achieve a minimum R_z_ at R_a_ = 2 µm, were considered. The most homogenous surface (a R_a_ to R_z_ ratio of about 0.2) was achieved at 90 °C after 6.26 h of etching. Etching at about 48 °C for 6.75 h, could also result in a homogenous surface with a 0.17 R_a_ to R_z_ ratio. Carvalho et al. showed that etching cpTi in 60% H_2_SO_4_ at 60 °C for 1 h, increased the surface isotropy of the machined surfaces from 17.4 to 91.5%^63^.

Surface roughness as one of the most important parameters that is effective in dental implant performance, have been studied in this research. Reports suggest that surface roughness is not the only effective surface parameter that determines the implant’s survival rate and osseous contact. Based on the clinical experiments, acid-etched titanium (SLA) promotes a greater and more rapid osseous contact when titanium-plasma-sprayed implants are used^33,64^. Surface hydrogen concentration and the formation of titanium hydride^65^, surface topology^44,66^ and surface wettability^67^ are some other parameters that are improved through the acid etching process.

In advanced dental implant surface treatment methods like SLA, a combination of air-abrasion parameters and acid etching variables can specify the final properties of the surface. After optimizing surface roughness by varying the acid etching parameters, we will study the sandblasting process. In future studies, we are interested in exploring the effect of different sand particle shapes on the surface configurations of dental implants. In addition, gas flow velocity, pressure, temperature, particle size, size distribution, and particle nature may also influence the final surface characteristics. However, since surface properties can be altered in the etching step, the outcomes of both treatments need to be considered to achieve the most favorable results.

## Conclusions

We evaluated the impact of etching solution temperature and etching process time on the surface characteristics (R_a_, R_z_, and WL) of dental implants in an acidic solution containing 48% H_2_SO_4_. The results showed that increasing both temperature and process time can enhance R_a_, R_z_, and WL. In addition, results confirmed the ability of an MLP-ANN model to predict the surface characteristics as a function of the etching parameters. Increasing the etching process time leads to higher R_a_, R_z_ and WL values. Based on the MLP-ANN predictions, increasing etching time at higher etching solution temperatures is more effective for improving the surface characteristics. In the following, NSGA-II based multi-objective optimization was used for obtaining the optimum time of etching and temperature of etching solution with the aim of minimizing the W_L_ and R_z_ and maximizing the R_a_. Finally, the results showed that the NSGA-II based optimization could be successfully applied for MLP-ANN based modeled etching process that could be used in dental implant surface treatment. Regarding the obtained results, ANN based models can be used for next studies of surface characteristics modelling. In addition, NSGA-II based multi-objective optimization can be successfully applied for prediction of the ideal operation condition, having the best surface.
